# Digital art as a novel medium for health communication: enabling interactive interventions and reconstructing health experiences in public health

**DOI:** 10.3389/fpubh.2026.1786916

**Published:** 2026-03-31

**Authors:** Rui-Li Guo, Yan Sun

**Affiliations:** 1 Department of Environmental Design, School of Fine Arts, Jiamusi University, Jiamusi, China; 2 Department of Arts and Crafts, School of Fine Arts, Jiamusi University, Jiamusi, China

**Keywords:** behavioral change, digital art, health communication, immersive experience, interactive intervention, interdisciplinary collaboration, public health

## Abstract

This perspective article explores digital art as an innovative medium for health communication. It argues that traditional health communication—often unidirectional and emotionally detached—frequently fails to support lasting behavioral change. In contrast, digital art introduces interactivity, immersive environments, and emotionally resonant narratives, enabling more engaging forms of health messaging and fostering deeper public involvement and awareness. Drawing on social cognitive theory, experiential learning, and media ecology, the article develops a conceptual framework and examines practical strategies such as narrative reconstruction, data visualization, and community-based co-creation. It also addresses key challenges, including issues of access, content accuracy, and ethical use of personal data in artistic health interventions. Looking forward, this perspective calls for stronger interdisciplinary collaboration, supportive policies, and evidence-based research to further integrate digital art into public health practice. By doing so, digital art could contribute meaningfully to more inclusive, participatory, and sustainable approaches to health promotion.

## 1 Introduction

Public health communication currently faces multiple challenges, including inefficiency in information dissemination, limited public engagement, and information overload in the digital era (1). These factors often hinder the effective delivery of health messages and contribute to public misunderstanding or apathy (1). Concurrently, digital art has emerged as a dynamic medium that integrates technology, aesthetics, and interactivity. It offers innovative possibilities for health communication by creating immersive and emotionally resonant experiences (2). Through its capacity to engage audiences on sensory and cognitive levels, digital art has the potential to transform how health information is conveyed and perceived, moving beyond traditional one-way communication models (3, 4).

Conventional health communication approaches are often constrained by their unidirectional structure, which limits opportunities for audience interaction and emotional connection (5). As a result, these methods may fail to foster meaningful engagement or sustained behavioral change (6). This study addresses two key questions: First, how can digital art, through interactive design, enhance the effectiveness of health communication (7, 8)? Second, in what ways can digitally mediated health experiences reshape public understanding and motivate proactive health practices (7, 9)? These questions highlight the need to explore new strategies that leverage artistic engagement to support public health objectives.

This perspective article aims to establish a theoretical and practical framework for the application of digital art in health communication. It seeks to identify how digital art can function as an effective medium for conveying health-related information and encouraging healthy behaviors. Furthermore, the research will examine specific use cases of digital art in public health contexts, such as health education, disease prevention awareness, and wellness promotion. From an interdisciplinary perspective—bridging art, technology, and public health—this work contributes to the evolving discourse on innovative health communication methods. Ultimately, it offers insights that can inform the design of more engaging, accessible, and impactful health interventions.

## 2 Related research and theoretical framework

### 2.1 Evolution and challenges of health communication

Health communication has progressively shifted from one-directional messaging toward participatory and interactive approaches (10). While traditional models effectively disseminated basic knowledge, they often fail to bridge the gap between awareness and lasting behavioral change (6). This “knowledge-action gap” persists partly because informational campaigns seldom engage audiences emotionally or motivate them to adopt and sustain new practices (11). The need for more engaging and immersive communication strategies has therefore become a central concern in contemporary public health.

### 2.2 Characteristics of digital art as a medium

Digital art introduces distinctive communicative possibilities through its core features: interactivity, immersion, and emotional resonance. Interactivity transforms audiences from passive receivers into active participants, enabling them to shape narratives and outcomes (12). Immersion—often supported by virtual or augmented reality—places users in simulated health scenarios, allowing for experiential learning in controlled settings (13). Furthermore, digital art employs multisensory design—visual, auditory, and sometimes tactile elements—to evoke empathy and connect with viewers on an affective level, complementing cognitive understanding with emotional engagement (14).

### 2.3 Theoretical framework

This study is grounded in an integrated theoretical framework combining social cognitive theory, experiential learning theory, and media ecology. Social cognitive theory explains how digital art can enhance self-efficacy through interactive simulation, allowing users to develop confidence in performing health behaviors (15). Experiential learning theory provides a process-oriented perspective, suggesting that digital art facilitates knowledge internalization through concrete experience, reflective observation, and active experimentation within immersive environments (16). From a macro perspective, media ecology helps conceptualize how digital art restructures the informational and relational landscape of health communication, creating new modes of engagement and meaning-making that can transform public interaction with health messages (17).

**Figure 1:**
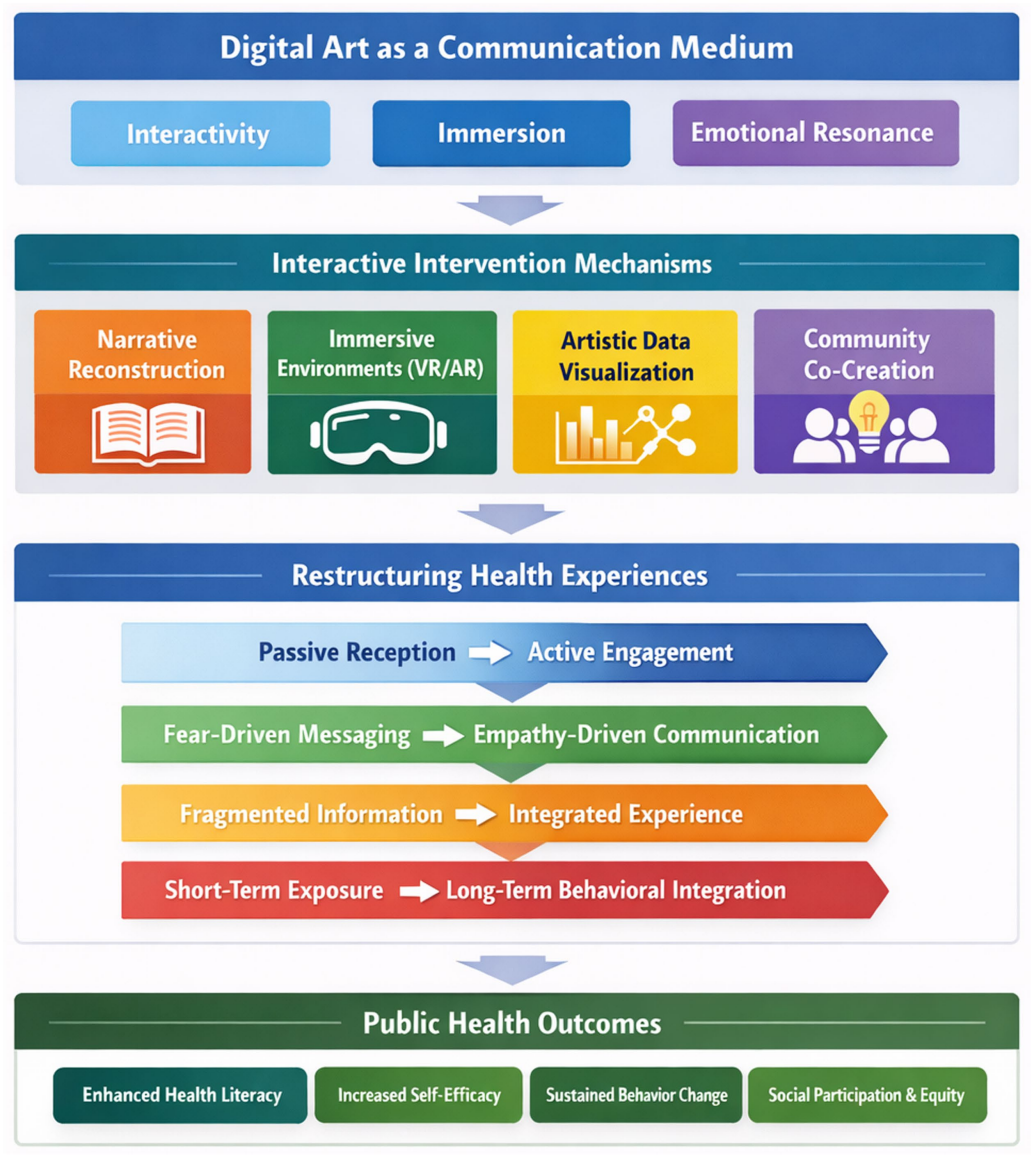
Conceptual framework of digital art-enabled health communication.

Together, these theories offer a multi-layered foundation for analyzing digital art’s potential to make health communication more experiential, effective, and participatory (Figure 1).

## 3 Interactive intervention mechanisms of digital art in health communication

Digital art enables health communication through multiple interactive intervention mechanisms, each targeting distinct experiential and behavioral pathways (Table 1).

**Table 1:** Mechanisms, applications, and public health implications of digital art–based health communication.

Digital art mechanism	Typical forms	Targeted experience change	Public health implication
Narrative reconstruction	Interactive storytelling, digital documentaries	Emotional resonance, empathy	Attitude change, motivation
Immersive environments	VR/AR simulations	Situated learning, self-efficacy	Skill rehearsal, preparedness
Data visualization	Artistic dashboards, generative visuals	Cognitive clarity, engagement	Health awareness, literacy
Community co-creation	Crowdsourced art, participatory platforms	Social identity, empowerment	Advocacy, collective action

VR, virtual reality; AR, augmented reality.

### 3.1 Narrative reconstruction: from information transmission to emotional resonance

Digital art reimagines health communication by transforming static information into dynamic, emotionally resonant narratives. Unlike traditional unidirectional models, it employs interactive storytelling to foster deeper engagement (18). For example, digital animations and interactive documentaries on topics such as cancer or mental health integrate personal experiences, social contexts, and medical insights, shifting audiences from passive recipients to active participants (18, 19). Personalized and non-linear narrative structures lower cognitive barriers, evoke empathy, and help individuals connect emotionally with health content (20). By translating statistics and clinical terminology into relatable human stories, digital art lays an emotional foundation for shifts in attitude and behavior (21).

### 3.2 Immersive environments: situated health experiences

Immersive technologies, including virtual reality (VR) and augmented reality (AR), enable situated health experiences that make abstract health concepts tangible and interactive (22, 23). VR can simulate scenarios such as disease outbreaks or public health crises, allowing users to witness the potential outcomes of their decisions in a controlled setting (23). AR applications overlay health guidance in real-world environments, turning everyday spaces into learning contexts (24). Grounded in embodied cognition—which posits that physical interaction shapes understanding—these experiences foster intuitive grasp of health causality. Through multisensory engagement and low-risk experimentation, users develop behavioral familiarity and enhanced self-efficacy, increasing their willingness to adopt and sustain health-promoting actions (22, 25).

### 3.3 Data visualization: artistic expression of complex information

As health-related data grow in scale and intricacy—from epidemic trends to indicators of chronic illness—conventional charts and graphs often lack public appeal and interpretive clarity. Digital art responds by transforming datasets into aesthetically engaging and narratively structured visual forms (26). Examples include generative artworks that animate real-time infection rates, or personalized visualizations that map individual health behaviors such as physical activity and sleep (27). This artistic approach improves cognitive processing, reduces interpretive effort, and elicits emotional responses through strategic use of color, movement, and form (28). By rendering data both accessible and meaningful, it sustains public attention and strengthens health awareness (29).

### 3.4 Community participation: collaborative creation and health advocacy

The participatory and scalable qualities of digital art support new modes of community involvement and collective advocacy in health communication. Online workshops and crowdsourced art projects invite the public to co-create and disseminate health-related content (7, 9). Initiatives focused on themes like mental health or chronic disease management do more than produce visual advocacy materials—they also foster shared identity and social cohesion among participants (30). In this way, artistic practice becomes a vehicle for health dialogue and a mechanism for social mobilization, strengthening community ties and channeling health awareness into organized action and mutual support (4, 31).

## 4 Pathways for digital art to restructure health experiences

### 4.1 From passive reception to active engagement

Digital art transforms health communication by shifting the audience role from passive recipients to active participants and co-creators (32). Through interactive works that respond to user choices and feedback, digital art allows individuals to simulate real-life health decisions in low-risk environments (33). This participatory design not only increases engagement and immersion but also enhances practical decision-making skills and self-efficacy (34, 35). The core of this transformation lies in empowerment—strengthening the user’s sense of agency and competence in managing health-related behaviors (36).

### 4.2 From fear-driven to empathy-driven communication

Conventional health messaging often relies on fear appeals, emphasizing the negative outcomes of unhealthy behaviors to motivate change (37). However, such approaches can provoke psychological resistance and avoidance, limiting their long-term effectiveness (37). Digital art offers an alternative by employing aesthetic and emotionally engaging experiences to inspire positive motivations such as empathy, hope, and curiosity (38). By visualizing the benefits of healthy living through compelling narratives, digital art fosters intrinsic motivation, turning health into an aspirational goal rather than a response to external threats (39, 40).

### 4.3 From fragmented information to integrated experience

While traditional health communication tends to present isolated facts, digital art synthesizes scientific knowledge, personal narratives, and cultural contexts into coherent, multisensory experiences (41). Through interactive games, animations, and immersive installations, it creates a unified environment where users can explore health topics in depth (9, 42).

Compared with text-dominant communication approaches, visually oriented digital art further strengthens this integrative process by reducing cognitive load and supporting more intuitive modes of information processing (43). Visual–narrative representations enable individuals to grasp complex health concepts through symbolic imagery, storytelling structures, and interactive engagement, thereby facilitating comprehension beyond linguistic or educational constraints (44). Such mechanisms are particularly beneficial for populations with limited health literacy, including older adults, individuals with lower reading proficiency, and culturally diverse communities (45). By combining aesthetic visualization with participatory interaction, digital art transforms abstract medical knowledge into accessible and emotionally meaningful experiences, helping to bridge communication gaps that often contribute to health inequities (26, 46).

By moving beyond information transmission alone, this integrative approach enables individuals to better understand not only what health behaviors are recommended but also why and how they can be incorporated into everyday life. Consequently, digital art contributes to a more holistic and personalized form of health literacy development, supporting deeper engagement and sustained behavioral understanding (41).

### 4.4 From short-term messaging to long-term behavioral integration

Beyond one-time campaigns, digital art can function as a sustainable health companion that encourages lasting behavioral change (47). Through mechanisms such as personalized data tracking, visual feedback loops, and gamified incentives, digital art creates ongoing interaction with users (48). These features help integrate health awareness into daily routines, supporting habit formation and maintenance.

In this context, visual feedback loops can be understood as adaptive interaction systems that continuously translate users’ behavioral or physiological data into real-time visual representations, allowing individuals to monitor progress, reflect on behavioral patterns, and adjust actions accordingly (49, 50). Rather than presenting static health information, wearable-integrated digital art platforms may transform indicators such as physical activity or sleep rhythms into evolving visual artworks, enabling intuitive perception of longitudinal health trends (50). Comparable approaches have been implemented in virtual reality–based lifestyle education programs in Europe and North America, as well as in community-oriented digital storytelling initiatives designed for adolescents and underserved populations (51–53). Importantly, current empirical evidence remains uneven across demographic groups, with relatively limited evaluation among older adults and populations in resource-constrained settings, highlighting a critical direction for future research and implementation (49, 53).

By embedding iterative feedback, experiential learning, and motivational reinforcement within daily life contexts, digital art effectively bridges the gap between health knowledge and long-term action, promoting sustained engagement rather than short-term compliance (54).

## 5 Challenges and ethical considerations

### 5.1 Technological accessibility and the digital divide

The implementation of digital art in health communication depends significantly on both technological access and digital literacy. This reliance may intensify existing inequities, particularly for groups with limited resources or technical skills—including older adults, low-income populations, and residents of underserved regions (55, 56). Overdependence on advanced or emerging technologies in health interventions risks undermining the public health principle of universal accessibility (57). It is therefore essential to prioritize inclusive design and equitable delivery. Practical approaches may include developing low-cost, intuitive digital tools; adopting accessible platforms such as mobile applications; and facilitating shared access through community hubs such as public libraries and health centers (58).

### 5.2 Accuracy of health information and artistic freedom

Digital art prioritizes creative expression, aesthetic engagement, and narrative flexibility, whereas health communication demands scientific accuracy, consistency, and clarity (59). This divergence can lead to tension: artistic interpretations may oversimplify or distort medical information, yet excessive adherence to factual rigor may reduce the emotional impact of the work (60). Achieving an appropriate balance between creativity and scientific validity therefore represents a central challenge in digital art–based health communication.

The rapid advancement of artificial intelligence (AI) is further reshaping digital art production within public health communication ecosystems. AI-generated images, narratives, and interactive environments offer substantial opportunities to enhance the scalability, adaptability, and personalization of health messaging (61). At the same time, these technologies may amplify existing concerns related to information accuracy, introducing additional risks such as algorithmic bias, misinformation propagation, and reduced transparency in content generation processes (62, 63). Without appropriate expert oversight, evidence verification, and validation mechanisms, AI-assisted artistic outputs may inadvertently disseminate inaccurate or misleading health information (61).

To address these challenges, structured interdisciplinary collaboration becomes increasingly essential. Involving artists, public health professionals, researchers, technologists, and data governance experts throughout the design, production, and evaluation stages can help ensure that creative innovation remains aligned with scientific rigor and ethical responsibility (64). Establishing interdisciplinary governance and quality-control frameworks will therefore be critical to balancing technological innovation with reliability, accountability, and public trust in digitally mediated health communication.

### 5.3 Privacy and data security

Many digital art projects in health rely on personal data—such as physiological metrics, activity logs, or location information—to create tailored user experiences. This practice raises important ethical concerns regarding privacy and data protection (65). From the initial design phase, developers should integrate strong safeguards, including transparent informed consent processes, clear communication about data use and retention, and robust technical measures such as data anonymization and encryption (66). Users should also retain control over their data, with options to access, modify, or delete their information as needed.

### 5.4 Complexity of impact evaluation

Assessing the effectiveness of digital art in health contexts presents methodological difficulties, as its influence often operates through emotional, cognitive, and behavioral pathways not easily captured by traditional metrics (67, 68). To address this, a mixed-methods evaluation framework is recommended. This should combine quantitative data—such as engagement metrics and usage patterns—with qualitative insights drawn from interviews, surveys, or reflective journals (69, 70). Longitudinal assessment can further help clarify the sustained impact of digital art experiences on health-related knowledge, attitudes, and behaviors over time (68, 69).

### 5.5 Environmental and sustainability considerations

The expansion of digital technologies in public health communication also raises important environmental and sustainability concerns. Digital infrastructures-including data centers, cloud computing, and immersive technologies-require substantial energy and water resources (71, 72). As digital art interventions scale globally, their ecological footprint warrants consideration alongside public health benefits (73, 74). The adoption of sustainable design strategies, such as energy-efficient platforms, low-resource technological solutions, and responsible data management practices, can help align digital innovation with broader environmental health objectives (74, 75).

## 6 Future directions: integrating digital art into public health

### 6.1 Expanding innovative applications

The continued integration of digital art in public health can advance across several practical domains. Two promising areas for development include tailored artistic interventions that respond to individual health profiles—for example, interactive digital environments designed to support emotional well-being and self-management in chronic care—and responsive digital art campaigns for public health emergencies (42, 76). In contexts such as climate health education or epidemic preparedness, visually immersive and emotionally resonant formats may strengthen public engagement, understanding, and adaptive behavior (77, 78).

### 6.2 Strengthening institutional and policy frameworks

To facilitate sustained collaboration, structured partnerships should be established between public health agencies, arts institutions, and technology sectors (79). Such partnerships can support co-creation, implementation, and rigorous evaluation of art-based health initiatives (80). Concurrently, public policy can play an enabling role through dedicated funding mechanisms and the integration of digital art projects into existing health promotion and cultural programs, ensuring both legitimacy and scalability (81).

### 6.3 Advancing research agendas

Future scholarship should prioritize two complementary lines of inquiry. First, longitudinal research is needed to examine the lasting effects of digital art interventions on health knowledge, attitudes, and behavioral outcomes (82). Such approaches are essential for moving beyond short-term engagement metrics toward a deeper understanding of durability and real-world public health impact. Beyond longitudinal investigation, future research should further examine the transferability of digital art–based health communication interventions across diverse socioeconomic and geographic contexts (83). Current evidence remains disproportionately concentrated in technologically advanced regions, while empirical studies conducted in low- and middle-income countries are still limited, despite the potentially substantial benefits of digital art approaches in settings where conventional health communication infrastructures may be constrained (84). Accordingly, comparative multi-setting research is needed to evaluate implementation feasibility, cultural adaptability, and equity-related outcomes across heterogeneous health systems (85). Second, cross-cultural comparative studies can help determine how digital art functions across diverse social, cultural, and technological contexts, informing the adaptation of interventions to local realities (86). Together, these research directions can build an evidence base to guide ethical, effective, and inclusive digital art applications in global public health practice.

## 7 Summary

Digital art enhances health communication by integrating interactivity, emotional engagement, and immersive experiences, effectively addressing the limitations of traditional informational approaches. By reshaping health narratives into participatory and embodied encounters, it fosters meaningful public involvement and promotes the internalization of healthy behaviors. This experiential framework positions interactive and emotionally resonant communication as a fundamental pathway to improving health outcomes.

This perspective article contributes a methodological advance to health communication through the deliberate integration of artistic, technological, and public health perspectives. It establishes a foundation for structured interdisciplinary collaboration and argues for recognizing digital art as a strategic component within public health systems. The findings advocate for its comprehensive inclusion to enhance the scope, relevance, and effectiveness of health promotion initiatives across diverse populations and settings.

## Data Availability

The original contributions presented in the study are included in the article/supplementary material, further inquiries can be directed to the corresponding author.
